# Out of the blue: the independent activity of sulfur-oxidizers and diatoms mediate the sudden color shift of a tropical river

**DOI:** 10.1186/s40793-023-00464-2

**Published:** 2023-01-19

**Authors:** Alejandro Arce-Rodríguez, Eduardo Libby, Erick Castellón, Roberto Avendaño, Juan Carlos Cambronero, Maribel Vargas, Dietmar H. Pieper, Stefan Bertilsson, Max Chavarría, Fernando Puente-Sánchez

**Affiliations:** 1 Institute of Microbiology, Technical University of Braunschweig, 38106 Brunswick, Germany; 2 Microbial Interactions and Processes Research Group, Helmholtz Centre for Infection Research, 38124 Brunswick, Germany; 3 Escuela de Química, Universidad de Costa Rica, San José, 11501-2060 Costa Rica; 4 Centro de Investigación en Ciencia e Ingeniería de Materiales (CICIMA), Universidad de Costa Rica, San José, 11501-2060 Costa Rica; 5 Centro Nacional de Innovaciones Biotecnológicas (CENIBiot), CeNAT-CONARE, San José, 1174-1200 Costa Rica; 6 Centro de Investigaciones en Productos Naturales (CIPRONA), Universidad de Costa Rica, San José, 11501-2060 Costa Rica; 7 Centro de Investigaciones en Estructuras Microscópicas (CIEMic), Universidad de Costa Rica, San José, 11501-2060 Costa Rica; 8 Deparment of Aquatic Sciences and Assessment, Swedish University of Agricultural Sciences, Lennart Hjelms Väg 9, 756 51 Uppsala, Sweden

**Keywords:** Geobiology, Hydroxyaluminosilicates, Hydrothermal, Sulfur oxidizing bacteria, Diatoms, Río Celeste

## Abstract

**Background:**

Río Celeste (“Sky-Blue River”) is a river located in the Tenorio National Park (Costa Rica) that has become an important hotspot for eco-tourism due to its striking sky-blue color. A previous study indicated that this color is not caused by dissolved chemical species, but by formation of light-scattering aluminosilicate particles at the mixing point of two colorless streams, the acidic Quebrada Agria and the neutral Río Buenavista.

**Results:**

We now present microbiological information on Río Celeste and its two tributaries, as well as a more detailed characterization of the particles that occur at the mixing point. Our results overturn the previous belief that the light scattering particles are formed by the aggregation of smaller particles coming from Río Buenavista, and rather point to chemical formation of hydroxyaluminosilicate colloids when Quebrada Agria is partially neutralized by Río Buenavista, which also contributes silica to the reaction. The process is mediated by the activities of different microorganisms in both streams. In Quebrada Agria, sulfur-oxidizing bacteria generate an acidic environment, which in turn cause dissolution and mobilization of aluminum and other metals. In Río Buenavista, the growth of diatoms transforms dissolved silicon into colloidal biogenic forms which may facilitate particle precipitation.

**Conclusions:**

We show how the sky-blue color of Río Celeste arises from the tight interaction between chemical and biological processes, in what constitutes a textbook example of emergent behavior in environmental microbiology.

**Supplementary Information:**

The online version contains supplementary material available at 10.1186/s40793-023-00464-2.

## Introduction

**Fig. 1:**
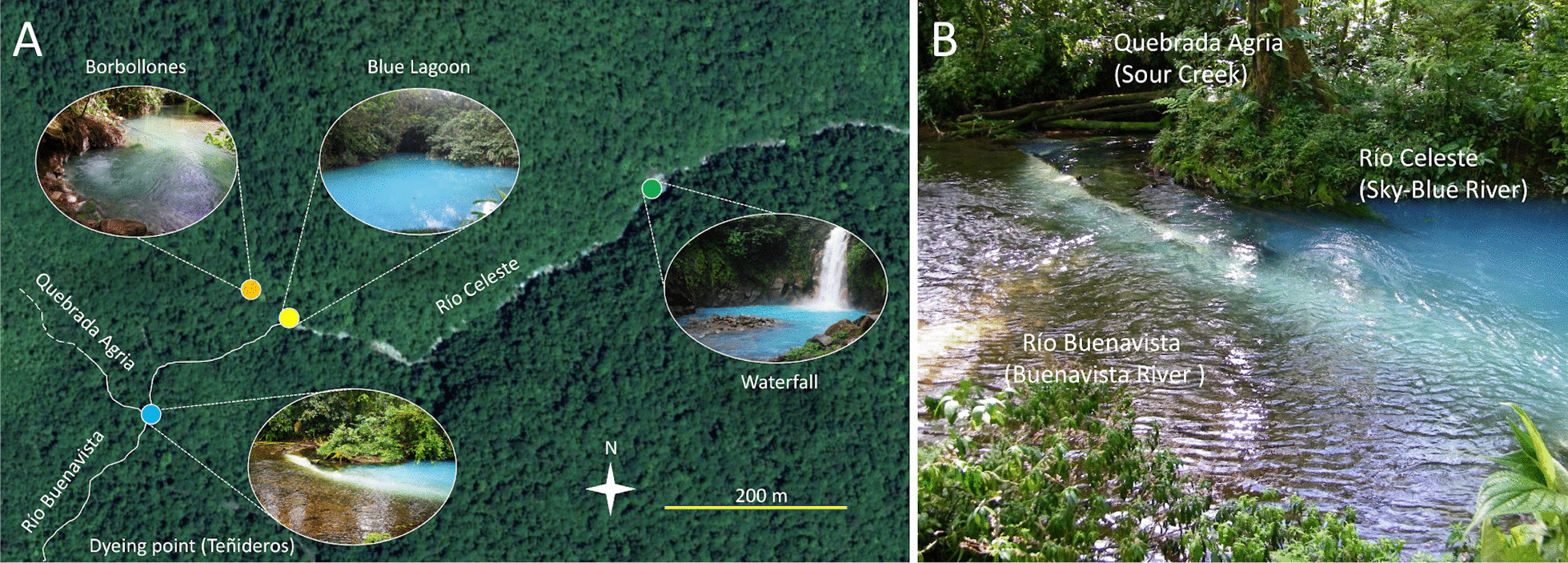
Environmental setting. **a** Satellite view of the Río Celeste area. River paths are shown with continuous lines when they are not directly evident from the satellite imagery. Dashed lines in Quebrada Agria indicate that the upstream of this river is still uncharted. **b** Detail of the ‘Teñidero’ (‘dyeing point’) confluence, showing the confluence between Río Buenavista and Quebrada, and the origin of the sky-blue river Río Celeste

Costa Rica is located in the Pacific rim of fire and therefore has a number of active volcanoes, and multiple manifestations of hydrothermal and volcanic origin such as thermal springs, acidic rivers or mineral-rich streams [1, 3]. One of the most striking manifestations of that activity, that can be observed in the complex basaltic-andesitic volcanic massif of Tenorio (Guanacaste Volcanic Mountain Range), is Río Celeste (“Sky-Blue River”). Located within the Tenorio Volcano National Park, this river is recognized as one of the most beautiful rivers in the world for its characteristic sky-blue color that contrasts with the dark surrounding rainforest (Fig. 1). Because of this, the river and the landscapes of the National Park have become an important hotspot for eco-tourism in Costa Rica [4] attracting more than 100,000 visitors per year [2, 5].

What makes Río Celeste unique is that it is possible to observe the specific site where the river turns blue. At this point known locally as ‘Teñidero’ (dyeing point), the two colorless streams Río Buenavista (Buenavista River) and Quebrada Agria (Sour Creek) merge, and the resulting waters quickly acquire the sky-blue color (Fig. 1). In a previous study, [6] showed that the sky-blue coloration of Río Celeste is caused by Mie light-scattering caused by particles suspended in its water stream. This phenomenon has been scarcely studied [7–9] and remains poorly understood. Because our previous measurements indicated that the particles present in Río Buenavista were smaller than the particles present in Río Celeste after the ‘Teñidero’ confluence [6], we initially proposed that the light-scattering particles originated via a pH-dependent aggregation mechanism when Río Buenavista (pH = 6.8) becomes acidified by Quebrada Agria (pH = 3.1), marking the beginning of Río Celeste (pH = 5.5). At that point we did not have measurements on the composition of the suspended particles, and we therefore alternatively examined the white material conspicuously deposited on the rocks which showed properties consistent with the presence of a poorly crystalline aluminosilicate mineral.

While our previous work demonstrated the role of light-dispersing aluminosilicates in giving Río Celeste its characteristic color, the formation mechanism of such particles was however not elucidated. Furthermore, our original study did not take into consideration the potential role of microorganisms in generating the chemical conditions conducive to this process. In the present study we provide a detailed analysis of the colloidal-sized particles that occur at the ‘Teñidero’ dyeing point, and examine the microbial communities that inhabit Río Buenavista, Quebrada Agria and the combined microbial community in Río Celeste. Our results challenge the previous perception in favor of a model where the particles causing Mie scattering mainly originate from dissolved aluminum and silicic acid coming from Quebrada Agria, with Río Buenavista acting as a secondary source of silicic acid and silica. Microbial community analyses show that Quebrada Agria hosts sulfur-oxidizing bacteria that are likely responsible for the acidic conditions in that source water, indirectly promoting aluminum and silicic acid mobilization. In contrast, Río Buenavista is a source of diatoms to Río Celeste whose presence influences silicon speciation and partitioning. Both the presence of sulfur-oxidizing bacteria in Quebrada Agria and the presence of diatoms in Río Buenavista are consistent with the chemical conditions of the respective stream. Our results shed new light on the combined biological and chemical processes that interactively cause formation of aluminosilicate particles that produce the beautiful sky-blue color that Río Celeste is well known for.

## Materials and methods

### Site description

The ‘Teñidero’ confluence (10°42′02.5″ N, 84°59′49.3″ W) is located in the Costa Rican Tenorio Volcano National Park, at the confluence of two streams: the neutral Río Buenavista and the acidic Quebrada Agria (Sour Creek). The whole area is embedded in the Volcanic Mountain Range of Guanacaste, and the region experiences frequent hydrothermal activity which gives rise to a multitude of niches such as thermal waters, acidic rivers or mineral-rich springs [2, 3]. Even though other rivers in Costa Rica show some sky-blue color, the ‘Teñidero’ stands out as a unique point of research and popular interest since only here the confluence of two seemingly colorless streams instantly turn into the sky blue-colored Río Celeste (Fig. 1).

### Sampling and field measurements

All necessary permits for sampling water and sediments were obtained from the National System of Conservation Areas (SINAC) of the Ministry of Environment and Energy (MINAE) of Costa Rica (Resolution No. 097-2014-ACAT). On 19 January 2014, samples of water and sediments were collected from Río Buenavista and Quebrada Agria about 5 m before their confluence (10°42′02.5″ N, 84°59′49.3″ W). Río Celeste’s samples were taken 20 m after the confluence. At each sampling point, 3 surface water samples (~ 10 cm depth, 1L each) and 3 sediment samples (between 10 and 50 g) were taken across the river. All samples for DNA analysis were collected in clean and sterile glass bottles, chilled on ice, stored at 4 °C, pooled and processed within less than 24 h. For qualitative analysis of diatoms, three surface water samples (~ 10 cm depth, 50 mL) were collected in 50-mL Falcon tubes for each stream, fixed and preserved with 1% Lugol’s iodine solution and 3% neutralized formaldehyde, and allowed to sediment for 24 h. Biofilm samples were also collected by brushing the surface of stones from the bottom of Río Celeste and preserved as described above. The chemical and physicochemical parameters for the three streams can be found at Additional file 1: Table S1 [6].

### Characterization of suspended materials

Water samples from Quebrada Agria, Río Buenavista and Río Celeste (1 L each) were individually filtered through porous membranes with a nominal pore size of 0.4 μm. The collected filters were allowed to dry at room temperature (20–25 °C). No residues were observed on the filter from Quebrada Agria. The residues collected from the water samples of Río Celeste and Río Buenavista were adhered to electrically conductive carbon tape for their analysis on a scanning electron microscope (SEM, Jeol JSM-IT500). This apparatus was equipped with a detector for energy dispersive X-ray spectroscopy (EDS) to determine the bulk chemical composition of the suspended materials collected in the filters. Infrared spectra of the suspended and deposited particulate materials from Río Celeste were measured with a Fourier-transform infrared spectrometer (Perkin Elmer, Spectrum 1000). The samples (10 mg) were mixed with anhydrous KBr (990 mg), finely ground using a mortar and pestle, and pressed to form discs containing the particles. With these discs, the spectra were obtained in the wavelength interval 400–4000 cm^−1^. Speciation calculations were carried out with PHREEQC [10]. For Río Buenavista, a measured alkalinity of 58.4 as bicarbonate was used.

For the precipitation experiments, water samples from Río Buenavista and Quebrada Agria (100 mL each) were respectively acidified with 0.005 M H_2_SO_4_ and neutralized with 0.01 M Na_2_CO_3_ until they reached the pH of Río Celeste (pH 5.5). Only the neutralization of the sample from Quebrada Agria produced suspended particles that were collected by centrifugation. The collected particles were resuspended in de-ionised water, centrifugated again and subsequently dried at room temperature. Such particles were pasted on carbon tape and analysed by SEM–EDS.

### Total DNA isolation, construction of 16S rRNA gene libraries and illumina sequencing

Three water samples (1L each) taken at different points along the river width were filtered through a vacuum filtration system under sterile conditions using a membrane filter (pore size 0.22 μm; Millipore, GV CAT No GVWP04700). To prevent filter rupture, a support membrane (pore size 0.45 μm; Phenex, Nylon Part No AF0-0504) was placed below. The upper filter was collected and stored at − 80 °C until processing. The DNA was extracted from aseptically cut pieces of the filter using the PowerSoil^®^ DNA Isolation Kit (MoBio, Carlsbad, CA, USA) as described by the manufacturer. Cells were disrupted by two steps of bead beating (FastPrep-24, MP Biomedicals, Santa Ana, CA, USA) for 30 s at 5.5 m s^−1^. For the construction of microbial 16S rRNA amplicon libraries, the V5-V6 hypervariable regions were PCR-amplified with universal primers 807F and 1050R [11]. Barcoding of the amplicons and addition of Illumina adaptors were conducted by PCR as described previously [12, 13]. The PCR-generated amplicon libraries were subjected to paired-end 2 × 250 Illumina MiSeq sequencing (Illumina, San Diego, CA, USA).

### Bioinformatic analysis of 16S rDNA amplicon data

Raw MiSeq sequences were quality-filtered and merged with *moira.py* v1.3.2 [14] with options*–paired–ambigs disallow–consensus_qscore posterior–qscore_cap 0–maxerrors 1–collapse True–alpha 0.005–output_format fastq*. A custom *python* script was used to expand the derreplicated files generated by *moira.py* so they could be used in subsequent analysis steps, and the contigs were then derreplicated and chimera screened with DADA2 v1.14.1 [15]. The resulting Amplicon Sequence Variants (ASVs) were aligned to the SILVA nr 132 reference database [16] using *mothur v1.44.0* [17]. The alignment was curated with *mothur* using the *screen.seqs* (options: *start* = *25,318, end* = *33,595, maxhomop* = *7, minlength* = *200, maxlength* = *275*) and *filter.seqs* (options: *vertical* = *T, trump* = *.*) commands, after which a UPGMA tree was constructed from the filtered alignment using *phangorn* v2.5.5 [18]. Variance-adjusted weighted UniFrac distances [19] were obtained from the tree with *GUniFrac* v1.1 [20]. Following McMurdie & Holmes [21], sequences were not rarefied prior to weighted UniFrac distance calculation. These distances were in turn used to compute a Non-metric Multidimensional Scaling with the *metaMDS* function from *vegan* v2.5.6 [22]. Further, we performed permutational multivariate analysis of variance [23] as implemented in the *PermanovaG* function from *GUniFrac* package to assess whether community composition was influenced by sample type (Water vs Sediment) and origin (Hydrothermally influenced vs Neutral). Finally, we used SINA [24] to taxonomically classify the ASV sequences, and SQMtools [25] to generate barplot figures. The following identity cutoffs to the closest match in the database were assigned to assign ASVs to the different taxonomic ranks [26]: phylum = 75%, class = 78.5%, order = 82%, family = 86.5%, genus = 94.5%. The taxonomy of each ASV is thus reported at the maximum resolution attainable without compromising a correct classification. Raw sequences were submitted to the sequence-read archive (SRA) under BioProject PRJNA747923. In order to be able to compare the composition of our samples to that of a known hydrothermally-influenced microbial community, we included the sequencing data from the hydrothermal spring Borbollones (located also inside the Tenorio Volcano National Park; Fig. 1) that was obtained in our previous study using the same sequencing methodology [3]. Reads assigned to Chloroplasts with SINA were further were annotated based on the naïve Bayesian classification with a pseudo-bootstrap threshold of 80% using RDP set18 [27]*.* Among them, those with more than 1000 total counts were manually annotated using the online Nucleotide BLAST tool from NCBI [28], and annotated against the Phyto REF database [29] using minimap2 [30].

### Identification of diatoms by optical and electronic microscopy

Both the water samples and biofilms were sedimented at room temperature for 24 h. The sedimented material was examined under a light microscope (Model IX-51, Olympus) at 40X or 100X optical magnification. Selected samples were analyzed in a scanning electron microscope (Model S-3700 N, Hitachi) using an accelerating voltage of 15 kV.

## Results

### Formation of minerals at the ‘Teñidero’ confluence

**Fig. 2:**
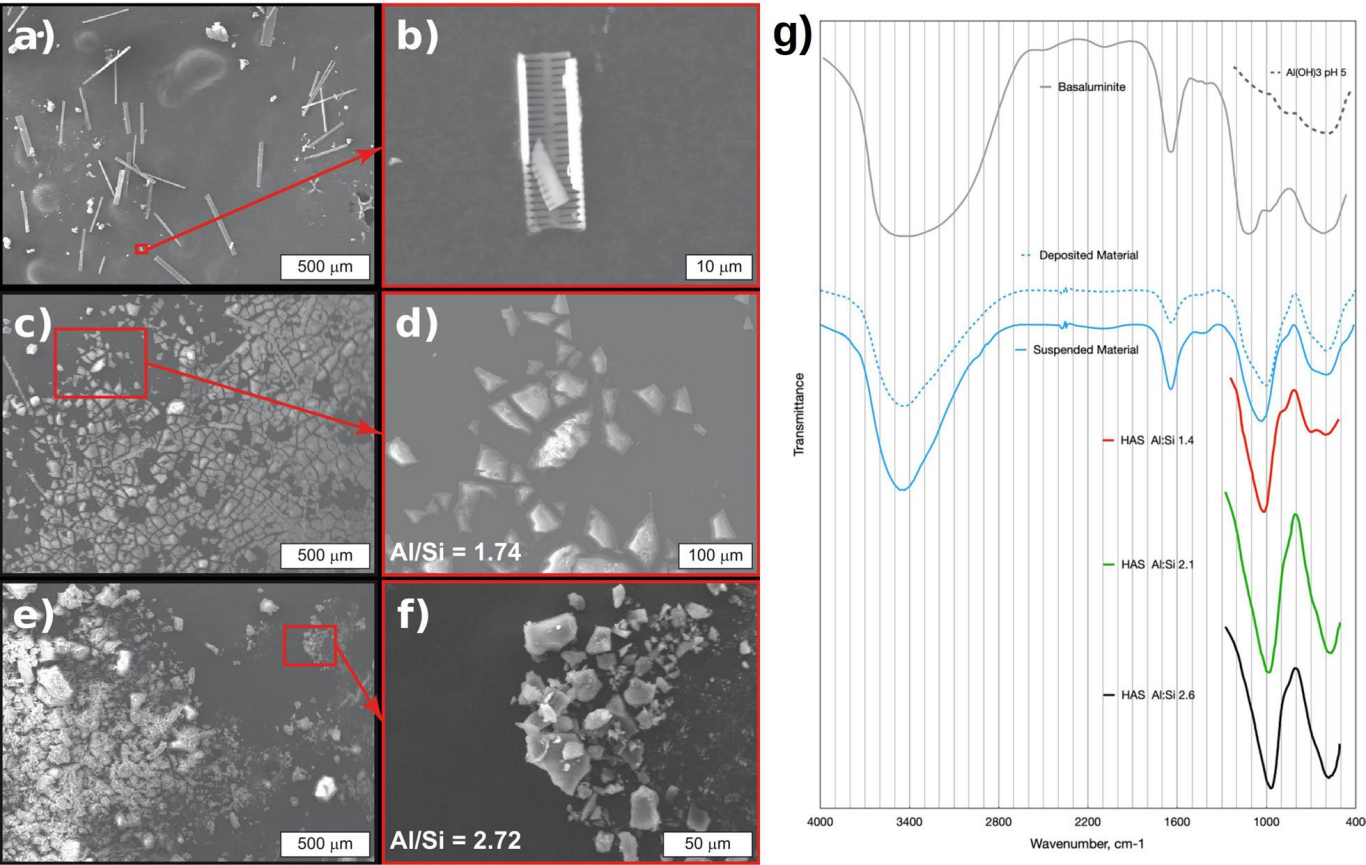
Chemical characterization of filtered or precipitated materials from the water streams at the confluence that originate Río Celeste. **a-f** Scanning electron microscopy (SEM) images from different materials The atomic Al/Si ratios were obtained by EDS scans. **a** Filtered materials from Río Buenavista; the solids retained by the membranes are mainly frustule fragments. **b** Detail of a frustule fragment. **c** Filtered materials from Río Celeste; frustule fragments along with agglomerated particles are retained by the filter membranes. **d** Magnification showing a detail of the agglomerated filtered particles from Río Celeste. **e** Agglomerated particulate materials obtained by in vitro precipitation of a water sample from Quebrada Agria. **f** Magnification performed on the specimen in **e**, showing the agglomerated particles. **g** Fourier-transform infrared (FTIR) spectrum of the particles suspended in Río Celeste, the precipitates occurring downstream from ‘Teñidero’, and other minerals known to precipitate in similar settings, including hydroxyaluminosilicates (HAS)

In order to determine the nature and origin of the light-scattering particles, we isolated the suspended material from the streams of Río Buenavista and Río Celeste, and analyzed them in bulk with scanning electron microscopy (SEM, Fig. 2), energy dispersive X-ray spectroscopy (EDS, Additional file 2: Fig. S1) and Fourier transform infrared spectroscopy (FTIR, Fig. 2g). The white deposits covering the rocks downstream from ‘Teñidero’ were also examined by FTIR.

Suspended matter in Río Buenavista consisted of particles that were too small to effectively scatter light according to the Mie principle, mixed with a few larger particles (e.g. frustule fragments) (Fig. 2a, b). Particles in Río Celeste River water formed larger aggregates in the filtered sample (Fig. 2c, d). A composition map assessed on a broad region (11.0 µm × 8.3 µm) of the solid from Río Celeste indicated the presence of aluminum, silicon and oxygen together with small and variable amounts of sulfur, with an Al/Si ratio of 1.74 (Additional file 2: Fig. S1). The FTIR spectrum of the particles in ‘Teñidero’ was found to resemble that of the deposits on the rocks downstream (Fig. 2g). Quebrada Agria lacked relevant suspended particles.

Acidification of Río Buenavista water did not produce any particles while the elevation of pH in Quebrada Agria waters produced abundant particles rich in aluminum, silicon and oxygen, with an Al/Si ratio of 2.72 (Fig. 2e,f).

### Microbial community composition at the Quebrada Agria (QA) stream

**Fig. 3:**
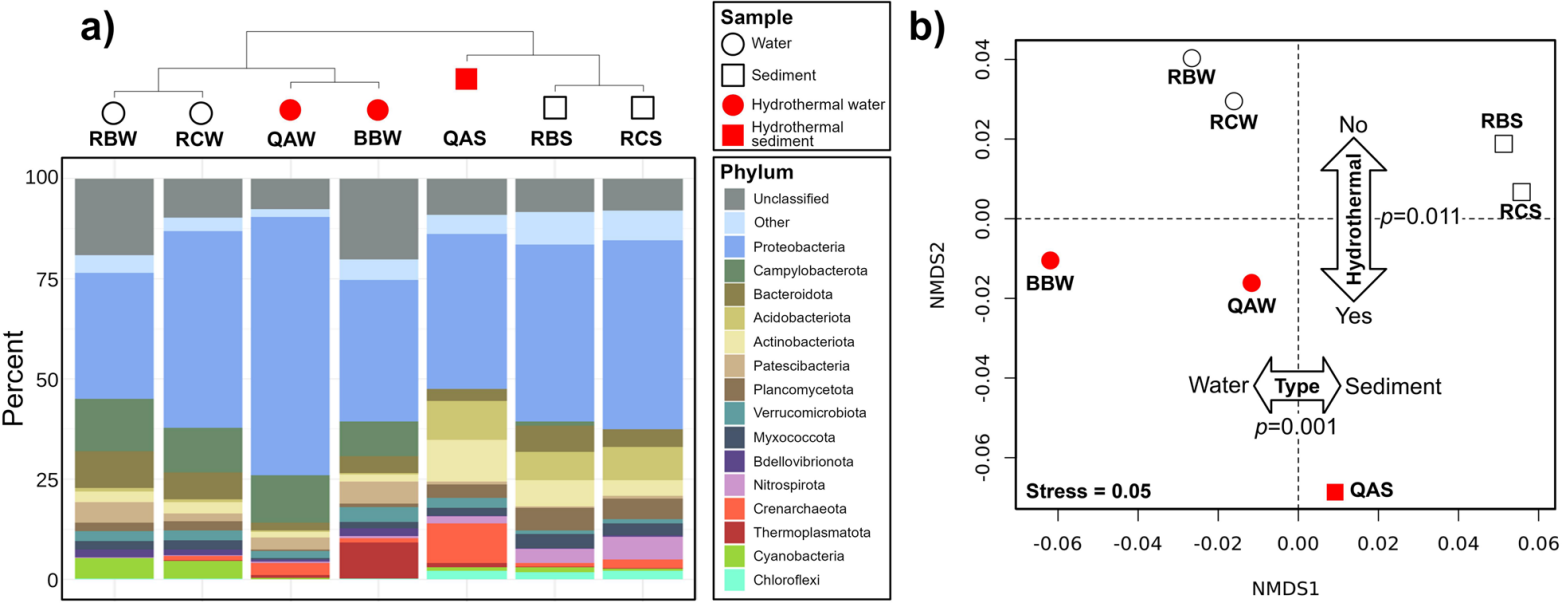
Microbial community composition around the ‘Teñidero’ confluence. Samples were from the two streams converging into the dyeing point (sample codes starting by RB and QA) and the resulting mixed stream (‘Río Celeste’, sample codes starting by RC). For each location, we analyzed plankton from the water (W) and sediments (S). An extra water sample from a nearby hydrothermal spring (BBW) was also included for comparative purposes. **a** Hierarchical clustering of the samples based on their variance-adjusted weighted UniFrac distances (top) and barplot showing their phylum-level composition (bottom). **b** Non-metric multidimensional scaling (NMDS) of the samples included in this study. Plotting symbol and color denote sample type (circle: water, square: sediment) and origin (white: neutral samples; red: hydrothermally-influenced samples). Two-way arrows and the associated p-values indicates a significant influence (PERMANOVA test using UniFrac distances) of sample type and origin in microbial community composition

**Fig. 4:**
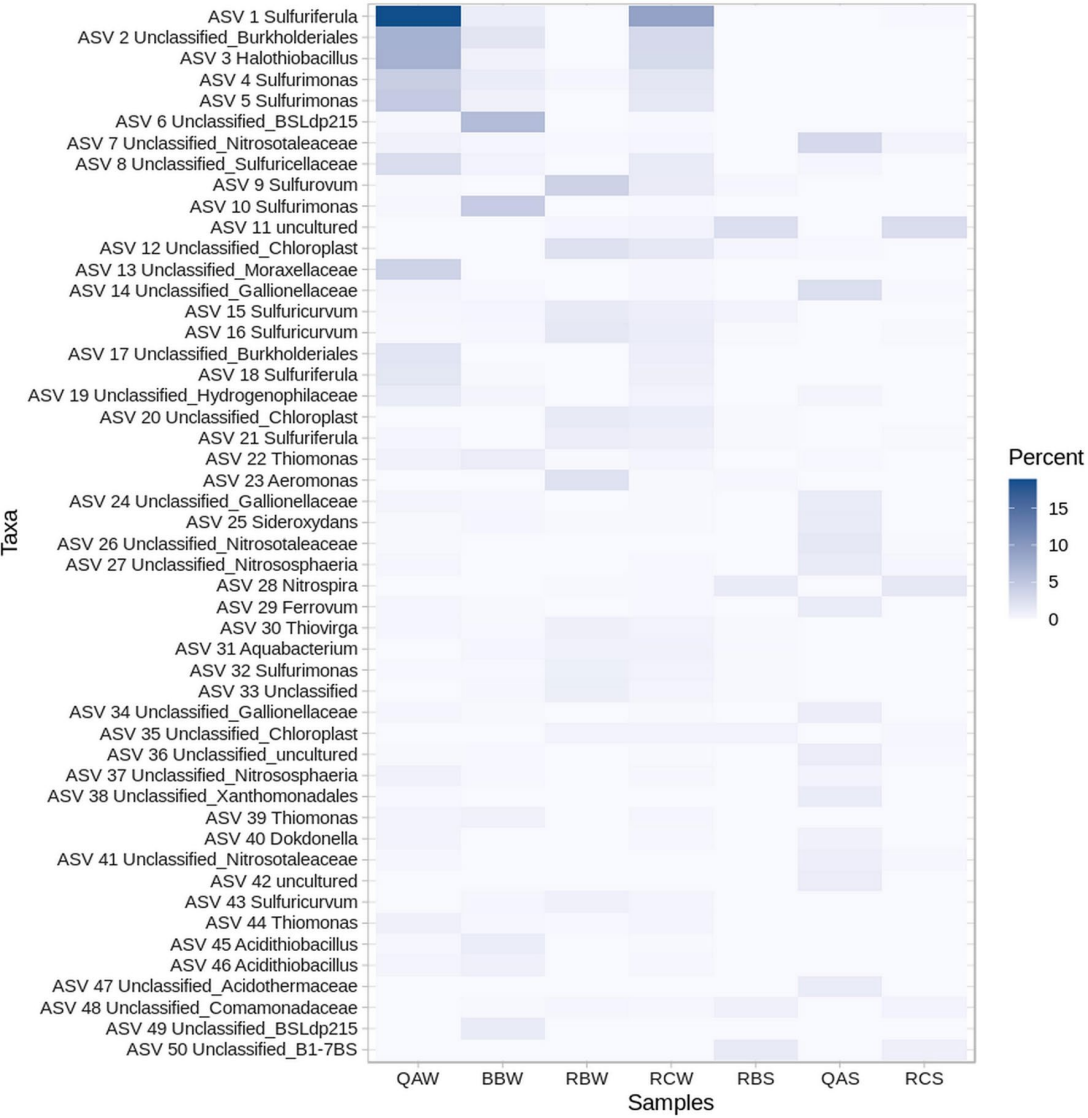
Distribution and taxonomy of the 50 most abundant ASVs. Heatmap intensity represents percentage abundance. The first two letters in the sample code indicate the river (QA: Quebrada Agria, BB: Borbollones, RB: Río Buenavista, RC: Río Celeste), the third letter indicates the material (W: water, S: sediment)

The microbial community in Quebrada Agria was dominated by Proteobacteria and Campylobacterota (Fig. 3a, QAW) assigned to known sulfur-oxidizing genera such as *Sulfuriferula* (Fig. 4, ASVs 1, 18), *Halothiobacillus* (ASV 3), *Sulfurimonas* (ASVs 4, 5), *Thiomonas* (ASV 44) or *Acidithiobacillus* (ASV 46). Archaea (Thaumarchaeota and Crenarchaeota) were also detected. Overall, the microbial composition of Quebrada Agria waters resembles to that of Borbollones (Fig. 3a, BBW, see hierarchical clustering), a nearby-located (at *ca* 300 m) hydrothermal spring whose microbial composition was recently described [3]. Overall, the sediment featured similar ASVs as the waters, but in different proportions (Fig. 3a, QAS). Campylobacterota were missing and Proteobacteria were present in lesser amounts and dominated by the iron-oxidizing family Gallionellaceae (Fig. 4, ASVs 14, 24), while the proportion of Archaea (Nitrosotaleaceae family, ASV 7, 26), Actinobacteria, Acidobacteria, Nitrospirota and Planctomycetota were higher. Some high-abundance ASVs were endemic to the QA sediments; this includes an Acidothermaceae actinobacterium (ASV 47) and a Nitrosomonadaceae gammaproteobacterium (ASV 81).

### Microbial community composition at the Río Buenavista (RB) stream

The most abundant phyla in Buenavista were Proteobacteria, Campylobacteriota and Bacteroidota (Fig. 3a, RBW). Among them, the most abundant bacterial genus was *Sulfurovum* (Fig. 4, ASV 9) followed by *Aeromonas* (Fig. 4, ASV 23). Many of the abundant bacterial taxa were related to the recycling of sulfur and nitrogen, such as *Sulfurimonas* (ASV 32)*, Thiovirga* (ASVs 30)*, Sulfuricurvum* (ASVs 43)*, Thiotrix* (Additional file 3: Table S2, ASV 53), Galliollenaceae (ASV 130) and Comamonadaceae (ASV 149). For eukaryotes, the second most abundant ASV in Buenavista’s waters was a Bacillariophyta (diatom) chloroplast (Fig. 4, ASV 12), which in general represented the majority of the cyanobacteria-related reads obtained in this study (Additional file 4: Fig. S2). Since the results obtained with SINA and RDP do not distinguish between different Bacillariophyta, we manually annotated the sequence of ASV12 using the online Nucleotide BLAST tool from NCBI [28]. The first hit was a 100% match to the chloroplast of the Bacillariophyceae diatom *Halamphora coffeaeformis* (NCBI accession number NC_044465.1), but several other perfect matches to other diatom species were also found (Additional file 5: Table S3). We also annotated it against the Phyto REF database [29], which classified it into the Cymbellaceae family. However, other abundant Bacillariophyta ASVs still had 100% matches to Phyto REF reference sequences from different orders (Additional file 5: Table S3), implying that the V5-V6 16S rRNA region amplified in this study is not good at discriminating between chloroplasts from different diatom groups. In order to achieve a better classification, we instead resorted to optical and electronic microscopy (see "Diatom composition at the Río Celeste (RC) stream" section). As for Quebrada Agria, the microbial population in Buenavista’s sediment was a mixture of endemic taxa and taxa which were also present in the water. Campylobacterota were at much lower relative abundances than in the water, and Actinobacteria, Proteobacteria, Acidobacteria, Nitrospirota and Planctomycetota increased (Fig. 3a, RBS). Among the taxa present in the sediments but not in the waters, the most abundant belonged to the Alphaproteobacteria and Gammaproteobacteria classes (Additional file 3: Table S2, ASVs 50, 58, 66, 86, 88) and the Bacteroidota and Nitrospirota phyla (ASV 185 and 120, respectively).

### Microbial community composition at the Río Celeste (RC) stream

The composition of Río Celeste’s planktonic microbiome was overall more similar to that of Río Buenavista than to that of Quebrada Agria (Fig. 3b), which is to be expected since Buenavista is the largest source of Celeste’s waters (*ca.* a 3:1 proportion with Quebrada Agria according to mass balance of conservative elements). The contribution of Quebrada Agria was still apparent from the high relative abundance of Proteobacteria (*Sulfuriferula;* Fig. 4, ASVs 1, 18) and Archaea. The sediment of Río Celeste was very similar to that of Río Buenavista, except for the higher presence of a few ASVs (Fig. 4, ASV 7–Nitrosotaleaceae; Additional file 3: Table S2, ASVs 61–Nitrospira and 74—Uncultured Planctomycetales) attributed to the influence of Quebrada Agria.

### Diatom composition at the Río Celeste (RC) stream

The analysis by optical- and electron-microscopy validated the results obtained by sequencing. A great variety of diatoms with different morphologies and sizes between 2 and 200 μm were observed (Additional file 4: Fig. S2). Morphologically, we identified organisms from the Bacillariophyta and Miozoa phyla. The identified species can be grouped into 3 classes (Bacillariophyceae, Mediophyceae and Dinophyceae) and 6 families (Diploneidaceae, Biddulphiaceae, Surirellaceae, Cymbellaceae, Catenulaceae and Peridiniaceae). Specifically, diatoms of genera *Diploneis*, *Biddulphia*, *Surirella*, *Cynbella*, *Amphora* were observed. The dinoflagellate *Peridinium* was also recorded being one of the most abundant taxa observed with light microscopy (Additional file 4: Fig. S2b).

## Discussion

### Hydroxyaluminosilicates are the main component of the minerals suspended in Río Celeste

Isolation of Río Celeste’s light-scattering particles by filtration through a 0.45 µm filter afforded a residue that we examined by SEM/EDX (Fig. 2c). The aggregates were larger than the diameters previously predicted by dynamic light scattering [6]; this can be explained by flocculation during filtering, drying and centrifugation, as it has been described previously [31], as well as by the removal of the smaller-sized particles during filtering. We thus find it safe to assume that the aggregates were representative of the light-scattering suspended materials.

We initially thought the precipitated particles, and the white deposits observed downstream, would be similar to those observed in the mixing of other acidic and neutral rivers and would consist of Al(OH)_3_ or the hydroxysulfates hydrobasaluminite or basaluminite [32, 33]. However, the composition of the particles suspended in Río Celeste was (in %) Al, 22.4; Si, 12.8; S 0.24; Fe 0.94, which not only contained a high amount of Si but also were too low in S compared to basaluminite’s 7% S. (Additional file 2: Fig. S1).

The low-frequency IR-spectra range of both the suspended particles and the rock deposits show only two main bands at 1000 and 605 cm^−1^ with shoulders at *ca.* 1100 and 680 cm^−1^ respectively. They are very different from basaluminite, which has strong IR bands at 606 and 1130 cm^−1^ [34]. (Fig. 2g) We could not gather enough suspended particles to obtain an XRD pattern but in our previous study the material deposited in the rocks showed a poorly crystalline material with broad peaks at 3.3 and 2.25 Å (Cu-K alpha, 2θ at 27 and 40°; see Additional file 2: Fig. S1 in [6]). This contrasts markedly with the 9.68 and 4.55 Å peaks of basaluminite [35]. Initially we could only match our XRD pattern to that of poorly crystalline aluminosilicates formed during sol–gel reactions and was the basis of our assignment. Other authors studying blue-colored lakes in Japan reached similar conclusions when studying the corresponding deposits of crystalline [9] or amorphous [36] aluminosilicate particles and suggested the amorphous particles have an “allophane-like” XRD pattern, with features at 3.4 and 2.3 Å similar to Río Celeste’s deposits.

Precipitation of aluminosilicate solids from solutions containing Al^3+^ and H_4_SiO_4_ starts at pH 5–6 when Al ions hydrolyze and begin forming gibbsite-like sheets that react with monosilicic acid to form amorphous aluminosilicate precursors [37]. In andosols these precursors might eventually form the Short-Range Ordered Aluminosilicates (SROAS) allophane and imogolite but also might give rise to very insoluble hydroxyaluminosilicates (HAS) [38, 39] with end-member compositions similar to those of allophane, (Al_2_O_3_)(SiO_2_)_1.3–2_·2.5–3 H_2_O or imogolite or kaolinite, Al_2_SiO_3_(OH)_4_. The first, named HAS_A_ forms if the concentration of Si(OH)_4_ is less than or equal to the total aluminum and has an Al/Si ratio close to 1. Where the initial concentration of Si(OH)_4_ is at least twice that of aluminum, further reaction with excess silicic acid yields HAS_B_ with a formula resembling imogolite and has Al/Si ratio close to 2. (Beardmore, 2016). For convenience, we will refer to the particles at the Teñidero as colloidal aluminosilicates which might consist of one or more of the above precursor phases, HAS or SROAS.

Due to their high insolubility the HAS are believed to be the main species controlling the solubility of aluminum in groundwater and in soils [40–42]. The IR spectra of all these SROAS show bands with frequency and relative intensity dependent on their Al/Si ratio and finally provided a good match and a way to characterize Río Celeste’s particles. [43–45]. Our sampled particles’ IR spectrum matches very well the asymmetric, high-frequency skewed bands of solids with Al/Si ratios around 1.4 as seen in Fig. 2g and compares well to Río Celeste’s particles 1.7 Al/Si ratio. The powder XRD patterns of these substances even match the 3.3 and 2.25 Å features found in Río Celeste deposits (compare Additional file 2: Fig. S1 in [6] with Fig. 2 in [45]).

### Chemical and environmental differences can explain precipitation of colloidal aluminosilicates in Río Celeste and precipitation of Basaluminite in other rivers.

Fast-mixing conditions at the Teñidero clearly produce a group of substances with structural and compositional properties similar to those of the HAS or SROAS. In contrast, in other studied bluish-water sites where acidic river water mixes with non-acidic tributaries, hydrobasaluminite and basaluminite are likely controlling aluminum solubility. Wanner et al. [34] characterized basaluminite deposits by IR spectroscopy at Ova Lavirun alpine creek. Water mixing calculations using PHREEQC were consistent with basaluminite controlling Al concentration as proposed for similar sites [32, 33]. At Colorado’s Paradise Portal site [46] the authors were able to examine the particles by TEM but they decomposed under the electron beam and did not yield an electron diffraction pattern. As the nanoparticles contained Al, S and Si their composition was assigned to hydrobasaluminite and amorphous silica based on their finding that hydrobasaluminite was the only aluminum phase identified in the fresh riverbed precipitates. Finally, in two Spanish acidic pit-lakes, hydrobasaluminite and schwertmannite were clearly detected along with kaolinite. However, the authors were careful to notice that incorporation of silica into these minerals could not account for all the SiO_2_ removal from water, and proposed that precipitation of proto-aluminosilicates would be consistent with the presence of kaolinite in the sediments [47].

In Additional file 6: Table S4 we show that Quebrada Agria has a much lower SO_4_/Al molar concentration ratio of 4.4 compared to the ratios in Ova Lapirum (19.9) and Paradise Portal (12.9) which could help explain why we do not observe sulfate materials like basaluminite. Our Si/Al concentration ratio of 3.0 is also higher, also consistent with formation of a more Si-rich phase. In Additional file 7: Note S1, we carried out PHREEQC speciation calculations of Río Celeste water at pH 5 using our analysis data and found HAS_B_ is actually expected to precipitate under those conditions. However, if we instead input the aluminum, sulfate and silicic acid concentrations from the precipitation sites of the Ova Lapirum and Paradise Portal sites into the speciation calculation, the saturation index for HAS_B_ becomes negative showing the effect of the different Al, SO_4_ and Si concentrations. Interestingly, the waters from the acidic pit lakes where hydrobasaluminite was found along with kaolinite also have low SO_4_/Al concentration ratios (5.5 and 6.3) close to that of Río Celeste. The waters show however a Si/Al ratio between that from Río Celeste and the two hydrobasaluminite-only precipitating sites making this is an intermediate case where both aluminosilicates and aluminum oxo sulfates form. Precipitation of specific Si and Al- containing mineral phases seems to have a strong dependence on element concentrations and speciation in the waters as well as local kinetic factors.

### Quebrada Agria (QA) is influenced by upstream hydrothermal activity

Quebrada Agria is a transparent and acidic stream (pH 3.1) largely devoid of suspended materials [6] while having a high content of dissolved sulfate (190 mg/L), chloride (71 mg/L), calcium (55 mg/L), and aluminum (12 mg/L) (Additional file 1: Table S1; [6]). While this distinctive chemistry suggests the influence of hydrothermal activity, the source of Quebrada Agria’s waters could not be determined during our sampling campaign and likely lies deep within the Tenorio Park rainforest. We therefore turned to the microbial community composition, and found that it was similar to that of the nearby (*ca* 300 m) Borbollones hydrothermal spring [3], sample code BBW). Samples from Quebrada Agria and Borbollones had a significantly different composition compared to the rest of the samples (Fig. 3b, PERMANOVA *p* = 0.011), and both shared prominent and functionally defined microbial taxa such as the sulfur-oxidizers *Sulfurimonas* and *Sulfuriferula* and the iron-oxidizer *Gallionela* (Additional file 3: Table S2). These genera have also been found in other hydrothermally influenced settings [52–54].

Based on these chemical and microbiological similarities, we hypothesize that Quebrada Agria is influenced by one or more upstream hydrothermal springs akin to the one described in [3]. Under this scenario, sulfide originating from hydrothermal fluids would be oxidized by microbial activity [55, 56], causing a decrease in pH and high sulfate concentrations observed near the ‘Teñidero’ site. The acidity of the water would in turn promote weathering and result in extensive mobilization of dissolved silicon and aluminum [33].

### Quebrada Agria is the main contributor to the generation of light-dispersing colloidal aluminosilicates

To further explore the origin of the particles, we performed an in vitro experiment in which water samples from the neutral Río Buenavista and the acidic Quebrada Agria were respectively acidified and neutralized to pH 5. Acidification of Río Buenavista water produced no noticeable changes upon reaching pH 5 indicating that a lowered Z-potential aggregation mechanism is not operating as we previously thought. However, a conspicuous proliferation of colloids was observed on the sample from Quebrada Agria upon taking it to pH 5 with sodium carbonate solution (Fig. 2c). This strongly suggests that, contrary to our original hypothesis, dissolved material from Quebrada Agria is the main source of the Río Celeste particles. The Al/Si atomic ratio in the synthetic particles was 2.7, higher than the 1.7 ratio observed in the naturally-occurring particles in Río Celeste. When bicarbonate alkalinity from Río Buenavista raises the pH of Quebrada Agria to the field-measured pH 5–6 values, precipitation starts and the high-silica river provides extra Si(OH)_4_ to the reaction thus avoiding partial formation of amorphous Al(OH)_3_ which could be thought to account for excess Al. It is believed that HAS form when silicic acid reacts with the growing Al(OH)_3_ gibbsite sheet. As we are observing the initial stages in the precipitation, it is conceivable that some Al(OH)_3_ could be present at some point. However, we only see evidence of its presence at small levels in the basification of Quebrada Agria experiment (Figure EDS) that produces a solid with a 2.7 Al/Si ratio. It is conceivable that the white rock-deposits downstream from the teñidero contain small amounts of other aluminum phases, accounting for excess aluminum in their composition (% Al, 33.8; Si, 10.7; S 2.40; Fe 4.99). Unfortunately, amorphous Al(OH)_3_ precipitated at pH 5 lacks the strong 1000 cm^−1^ IR band and only shows a broad band at 570 cm^−1^ not detectable in our IR spectrum amongst other phases (Fig. 2g). Furthermore, when precipitated at pH 5, Al(OH)_3_ has an essentially featureless XRD pattern and will not show in our XRD measurements [48] that only have features assignable to poorly crystalline aluminosilicates.

At the Teñidero, particles form within a few seconds and the resulting light-scattering stable colloidal phases are visible at least 14 km downstream. Smaller particles from Río Buenavista [6], probably become embedded with the precipitate at the Teñidero accounting for its smaller 1.7 Al/Si ratio and iron content. The chemistry of the Teñidero then bears some resemblance to that of flocculation water treatment processes that adsorb clay particles on positively charged Al/Si flocs prepared by reaction of aluminum potassium sulfate (alum) and silicic acid at pH 5 and above [49–51]. Our work is only concerned with the initial colloidal phase where the transformation of aluminum hydroxide into HAS or even SROAS is starting to take place. Enough dissolved species do remain in Río Celeste water and additional mineral phases are likely to form downstream but are not the focus of this report.

### Río Buenavista (RB) is a circumneutral stream with standard chemical composition

The Río Buenavista has many of the features typically seen in freshwater ecosystems: pH is close to neutral (6.8) and the chemical composition of the water is within the established levels of metals and anions suitable for human consumption (Additional file 1: Table S1). The river is largely transparent and shows no noticeable suspended particles but do contain silica particles with an average diameter of 184 nm [6].

The most abundant ASV in Buenavista’s waters were assigned to the *Sulfurovum* genus (Fig. 4, ASV 9). Other genera related to sulfur cycling such as *Sulfurimonas, Thiovirga* or *Thiotrix* were also present in moderate amounts*.* The dominance of *Sulfurovum* is somewhat puzzling, as this genus is usually found in hydrothermal vents [52], cold seeps [57], groundwater aquifers [58], freshwater sediments [59] or as endosymbionts of larger organisms [60]. It is unclear if any of these conditions would apply in Río Buenavista waters: they could be originating from the sediments (where they are also present, albeit at lower relative abundance), be indicative of hydrothermal activity (although Buenavista’s waters have an apparently standard chemical composition), or originate from some nearby groundwater seep.

The second most abundant bacteria (third most abundant ASV including non-bacterial sequences) was an ASV from the heterotrophic *Aeromonas* genus (Fig. 4, ASV 23). *Aeromonas* is commonly seen in freshwaters and drinking waters [61], and appears either as a free-living organism or associated to crustaceans or fish, for which they can act as pathogens [62]. The abundance of this genus in freshwaters has been considered an indicator of trophic status, analogous to Secchi disc depth, total phosphorus, or chlorophyll *a* [63]. Their high relative abundance suggest that Río Buenavista is somewhat eutrophic.

Finally, the second most abundant ASV in Río Buenavista’s waters was classified as a Bacillariophyta (diatom) chloroplast (Fig. 4, ASV 12). Manual search against the Phyto REF database indicated that the chloroplast 16S rRNA gene sequence was affiliated to the Cymbellaceae family. (Additional file 5: Table S3). This was confirmed by light and electron microscopy, which revealed the presence of the diatom families Diploneidaceae, Biddulphiaceae, Surirellaceae, Cymbellaceae, Catenulaceae and Peridiniaceae (Additional file 4: Fig. S2)*. Cymbellaceae* (found both by amplicon sequencing and microscopy) have been reported in other tropical rivers [64–66] including rivers from Costa Rica [67]. Other diatom taxa commonly reported in these rivers, such as *Nitzschia* or *Gomphonema* [64, 65, 67, 68] were however not detected in our study. We note that the abundance of diatoms in Río Buenavista might be much higher than suggested by the relative abundance of ASV 12, since our sample processing protocol was not optimized for retrieving DNA from hard-to-lyse eukaryotic cells. Our observations are nonetheless consistent with previous reports of high diatom activity in tropical rivers with low turbidity [69].

### Diatoms as sources of colloidal silica in Río Buenavista

Diatoms incorporate dissolved silicate into their frustules, being the main source of biogenic silica in riverine settings [70, 71] and under some circumstances accounting for the majority of the total silicon budget [70, 72]. Most of the biogenic silica in rivers is not actually associated to living diatoms, but to detrital particles that originate when diatoms die or are preyed upon [73, 74]. Interestingly, diatom cultures have been shown to produce extracellular silica nanoparticles in the 150–400 nm diameter range [75], which is compatible with the 184 nm average diameter of the particles found in Río Buenavista [6]. Lithogenic silica (coming from the weathering of minerals such as clays, silts and sand) can also represent a large fraction of the total silicon in rivers [76]. The particle size from such processes is however in the micrometer scale [77, 78], much larger than the particles detected here [6]. Additionally, Río Buenavista was transparent and devoid of apparent particulate material. Overall, the data are consistent with the notion that diatoms, rather than lithogenic sources, are responsible for the colloidal silica present in the water from Río Buenavista. Diatom activity would therefore control the partitioning of silicon between the dissolved and colloidal forms in Río Buenavista, making them indirect contributors to the Río Celeste phenomenon.

### Biological influence in the distinctive color of Río Celeste

**Fig. 5:**
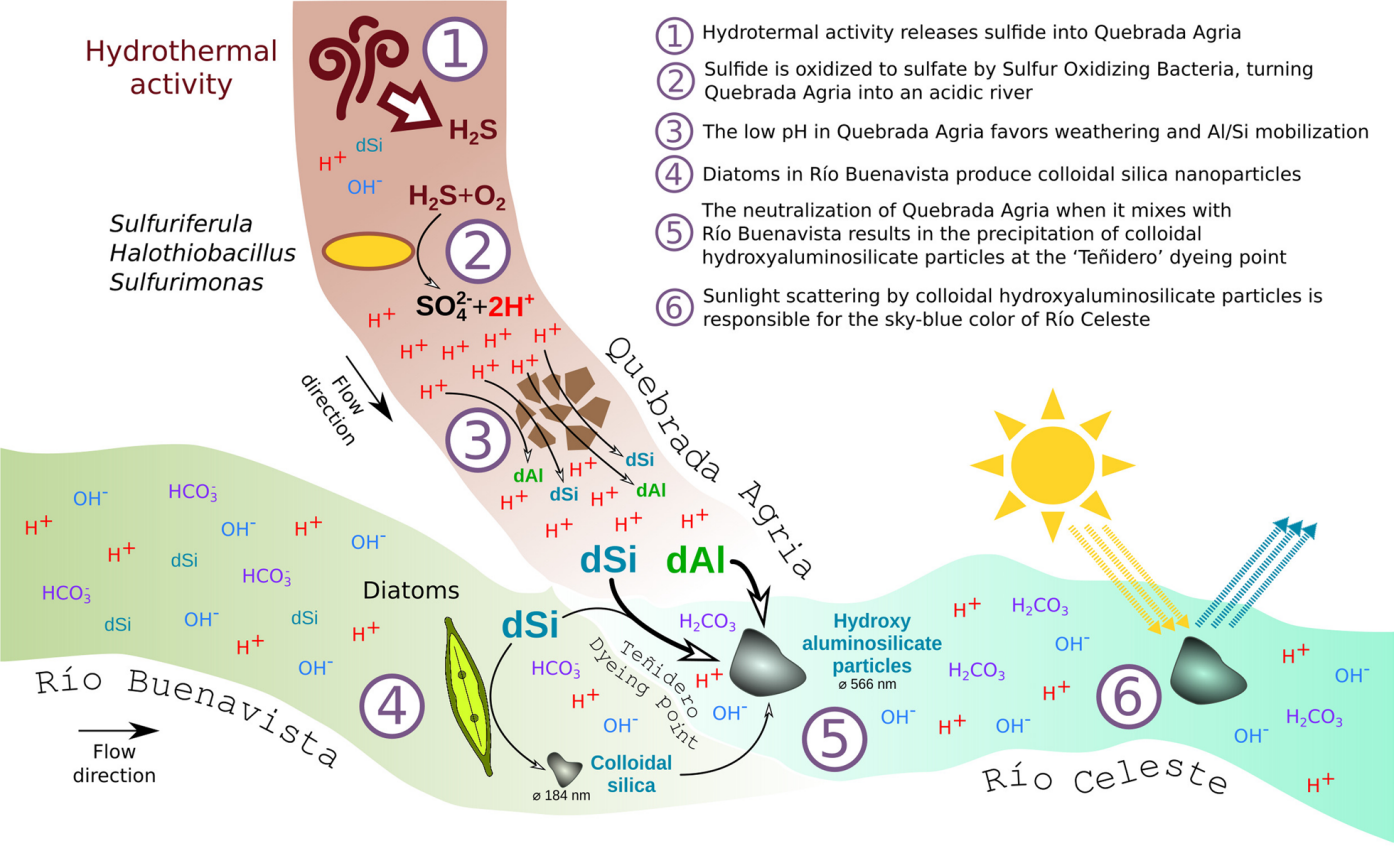
Conceptual biogeochemical model of the origin of Río Celeste’s sky-blue color

Río Celeste arises at the ‘Teñidero’ confluence, when the acidic and aluminum-rich Quebrada Agria mixes with the neutral Río Buenavista. Our experiments suggest that the partial neutralization of Quebrada Agria initiates a precipitation reaction in which aluminum and silicic acid precipitate with dissolved silica and likely incorporate also smaller particles coming from Río Buenavista, resulting in colloidal hydroxyaluminosilicates that scatter light as described in [6]. The interaction of two streams with a characteristic physicochemical composition is thus necessary for the emergence of the unique Río Celeste phenomenon. Our data strongly suggest that, in both streams, this composition is biologically mediated (Fig. 5).

In Quebrada Agria, hydrothermal sulfide is oxidized by bacteria such as *Sulfuriferula and Sulfurimonas* resulting in an acidic fluid capable of mobilizing silicates and metals such as aluminum, as has been described for other ecosystems [33, 55, 56]. Meanwhile, the partitioning of silicon in Río Buenavista seems to be controlled by diatoms, which are in turn the most likely source for the colloidal silica particles suspended in the stream. Diatom particles are known for their ability to act as flocculants [79, 80], and incorporate aluminum both during biogenesis [81] and afterwards [82, 83], leading to deposition of aluminosilicates in riverine systems [84]. Diatoms might therefore be facilitating the chemical precipitation of minerals at ‘Teñidero’, as previously described for other system [85], resulting in the precipitation of particles of the right size for a more intense blue coloration.

### Conclusions

The sky-blue color of Río Celeste originates at the interface between chemistry and biology, and thus represents a textbook example of emergent behavior in environmental microbiology: by themselves, the biogeochemical processes operating in Quebrada Agria and Río Buenavista are fairly common in nature, but when put together they give rise to what could be described as a small natural wonder. Río Celeste is of relevance not only from the geobiologist’s point of view, but also for its socioeconomic impact in the Costa Rican eco-tourism industry. This unique phenomenon might however be fragile: the coloring of the river depends on just the right combination of factors, and this delicate equilibrium could very well be threatened by human activity and environmental change. Further research is therefore required in order to characterize the nature and temporal dynamics of its chemical and microbiological components, and assess its vulnerability to biotic and abiotic stressors.

## Supplementary Information

**Additional file 1. Table S1** Chemical composition and physicochemical properties of the three streams studied in this work. Data taken from [6], DOI: 10.1371/journal.pone.0075165.

**Additional file 2. Fig. S1** Energy dispersive X-ray spectroscopy (EDS) of the particles shown in Figure 2

**Additional file 3. Table S2** Sequence, abundance distribution and taxonomy of the Amplicon Sequence Variants (ASV) detected in this work. Sequences are annotated with SINA, Chloroplast sequences were further annotated with RDP.

**Additional file 4. Fig. S2** Presence of diatoms around the ‘Teñidero’ confluence. a) Relative abundance of cyanobacteria-like ASVs in the different samples. Most of the Bacillariophyta ASVs could be further assigned to different diatom groups (Additional file 5: Table S3). Sample codes are described in the legend for Figure 3b, b) Light microscopy image from a Río Celeste sample showing the presence of diatoms with different morphologies, c) Light microscopy image from a Río Celeste sample showing the presence of the dinoflagellate Peridinium, d-f) Electronic microscopy images showing details on diatom frustules.

**Additional file 5. Table S3** Closest NCBI nr and Phyto REF matches of the most abundant (more than 1000 total counts) Chloroplastic ASVs. For each ASV, we include its best hit in the nr database to a sequence belonging to named species, as well as their best hit in the Phyto REF da-tabase. In case two or more hits were tied at the best score, all of them are included

**Additional file 6. Table S4** Concentration of selected chemical species in waters where aluminum minerals precipitate.

**Additional file 7. Note S1** Parameters used in PHREEQC speciation calculation.

## Data Availability

Raw sequences were submitted to the sequence-read archive (SRA) under BioProject PRJNA747923.
