# Supplementary figures and images for: Out of the blue: the independent activity of sulfur-oxidizers and diatoms mediate the sudden color shift of a tropical river
Source: Environ Microbiome. 2023 Jan 19;18:6. doi: 10.1186/s40793-023-00464-2 (PMC9854191; doi:10.1186/s40793-023-00464-2)

## Río Buenavista

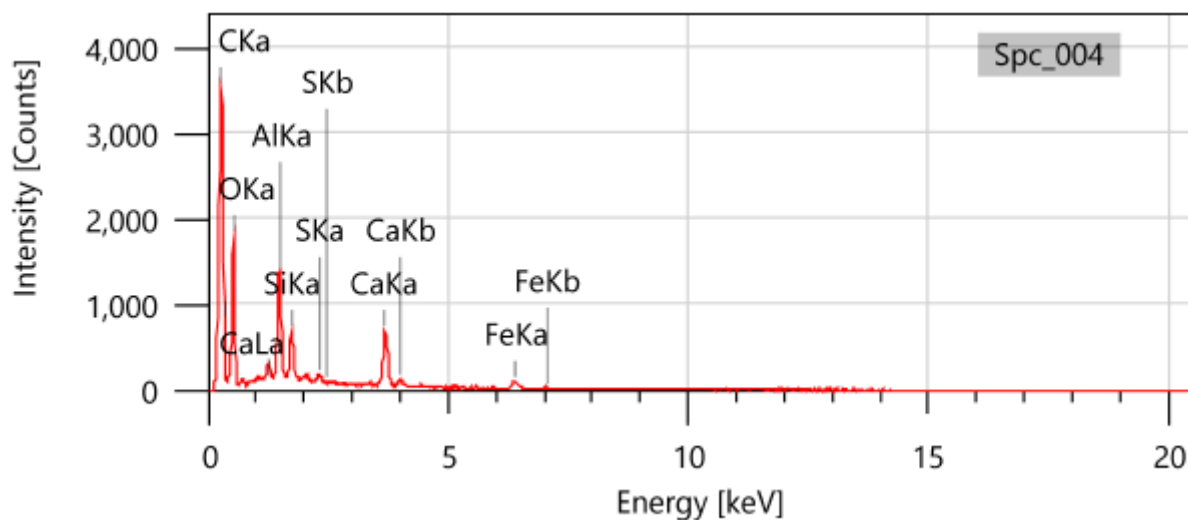

## Río Celeste

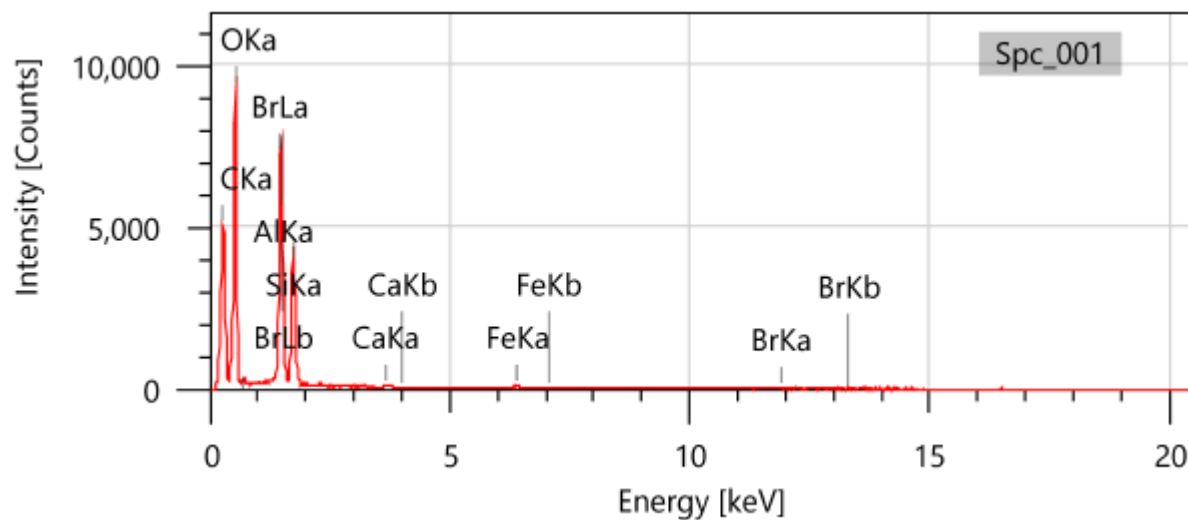

## Quebrada Agria (neutralization experiment)

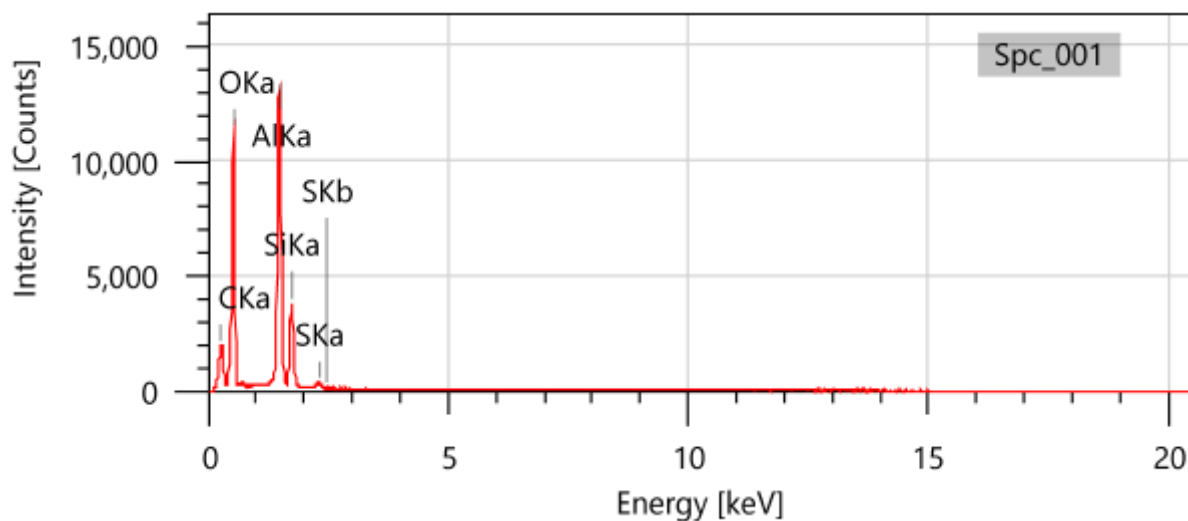

Supplement: Supplementary file 2 — Additional file 2. Fig. S1 Energy dispersive X-ray spectroscopy (EDS) of the particles shown in Figure 2 [file 40793_2023_464_MOESM2_ESM.pdf]

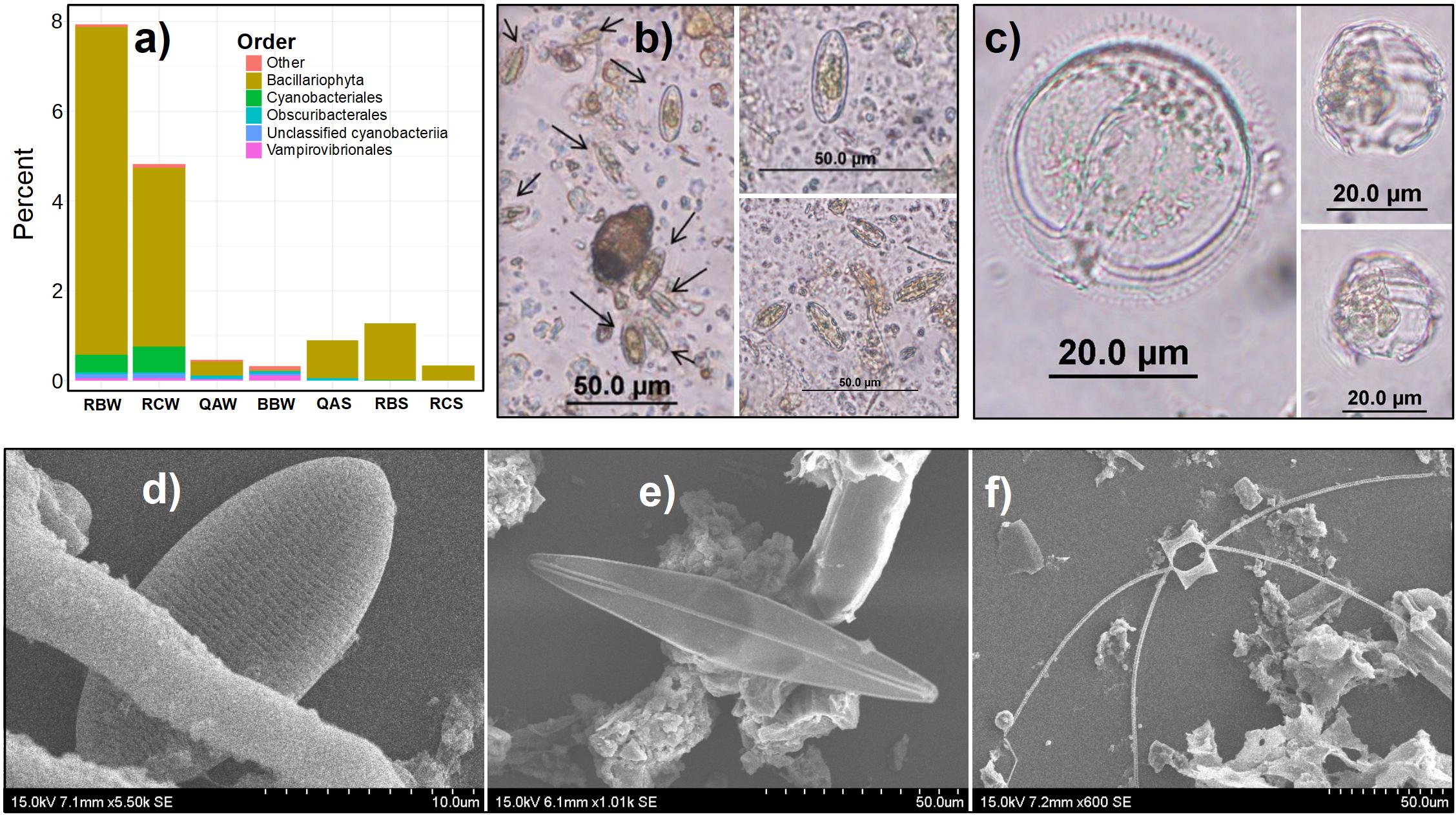

Supplement: Supplementary file 4 — Additional file 4. Fig. S2 Presence of diatoms around the ‘Teñidero’ confluence. a) Relative abundance of cyanobacteria-like ASVs in the different samples. Most of the Bacillariophyta ASVs could be further assigned to different diatom groups (Additional file 5: Table S3). Sample codes are described in the legend for Figure 3b, b) Light microscopy image from a Río Celeste sample showing the presence of diatoms with different morphologies, c) Light microscopy image from a Río Celeste sample showing the presence of the dinoflagellate Peridinium, d-f) Electronic microscopy images showing details on diatom frustules. [file 40793_2023_464_MOESM4_ESM.jpg]
