# Supplementary material for: Out of the blue: the independent activity of sulfur-oxidizers and diatoms mediate the sudden color shift of a tropical river
Source: Environ Microbiome. 2023 Jan 19;18:6. doi: 10.1186/s40793-023-00464-2 (PMC9854191; doi:10.1186/s40793-023-00464-2)
Supplement: Supplementary file 7 — Additional file 7. Note S1 Parameters used in PHREEQC speciation calculation. [file 40793_2023_464_MOESM7_ESM.pdf]

Input file for Río Celeste PHREEQC speciation calculations.

Literature data were taken from the literature for Hydrobasaluminite [Sánchez-España *et al.,* 2011] and HAS-B [Dobrzyński 2006, 2007]

```
TITLE Celeste pH 5.00
SOLUTION 1 Celeste
        units   ppm
        pH      5.00
        pe      4
        density 1.004
        temp    22.0
        redox   O(0)/O(-2)
        S(6)  104
        Cl    31
        F           0.65
        Na    10
        K           2.2
        Ca    32
        Mg    7
        Al    5
        B           0.57
        Si    29 as Si
        Sr    0.13
        Mn    0.28
        O(0)    9.15  O2(g) -0.7
        C(4)    1.0  CO2(g) -3.5
PHASES
       HAS-B
          Al2Si2O5(OH)4 + 6 H+1 = 2 Al+3 + 2 H4SiO4 + H2O
          log_k     14.0

     Hydrobasaluminite
        Al4(SO4)(OH)10:12 H2O + 10 H+1 = 4 Al+3 + SO4-2 + 22 H2O
        log_k     23.9

END
```

I order to do a comparative Speciation Calculation the Input File was modified using the data below. HAS_B precipitation would not be expected if sulfate, aluminum and silicic acid concentrations at Rio Celeste were similar to those at the low Si concentration sites Ova Lapirum OL9 and Paradise Portal PP3 precipitation sites.

Input Data for Speciation Calculations:	**Rio Celeste** (S(6) 104, Al 5, Si 29 as Si)	**Ova Lapirum OL9** (S(6) 165, Al 1.8, Si 3.1 as Si)	**Paradise Portal PP3** (S(6) 576, Al 4.2, Si 8.3 as Si)
Hydroxyaluminosilicate B Saturation Index	0.87	-2.52	-1.36
Hydrobasaluminite Saturation Index	4.62	1.94	3.04

Sánchez-España, J.; Yusta, I.; Diez-Ercilla, M. Schwertmannite and Hydrobasaluminite: A Re-Evaluation of Their Solubility and Control on the Iron and Aluminium Concentration in Acidic Pit Lakes. *Applied Geochemistry* **2011**, *26* (9–10), 1752–1774.

Dobrzyński, D. Chemistry of Neutral and Alkaline Waters with Low Al3+ Activity Against Hydroxyaluminosilicate HASB Solubility. The Evidence from Ground and Surface Waters of the Sudetes Mts. (SW Poland). *Aquat Geochem* **2007**, *13* (3), 197–210.

Dobrzyñski, D. Silica Solubility in Groundwater from Permian Volcanogenic Rocks (the Sudetes Mts., SW Poland) — the Role of Reversible Aluminosilicate Solids. *Geological Quarterly* **2006**, *50* (4), 407–417.
